# 643 Burn Unit Trends in Cannabis Use and Associations with Complications and Outcomes

**DOI:** 10.1093/jbcr/iraf019.272

**Published:** 2025-04-01

**Authors:** Punit Vyas, Arman Fijany, Emily Swafford, Tyler Murphy, Jordan Garcia, Robel Beyene, Stephen Gondek, Anne Wagner, Elizabeth Slater

**Affiliations:** Vanderbilt University Medical Center; Vanderbilt University Medical Center; Vanderbilt Burn Center; Vanderbilt Burn Center; Vanderbilt University Medical Center; Vanderbilt University Medical Center; Vanderbilt University Medical Center; Vanderbilt University Medical Center; Vanderbilt University Medical Center

## Abstract

**Introduction:**

Prior drug use and intoxication often complicate inpatient admissions. Studies have established an increasing rate of cannabis use in burn unit admissions in recent years, likely related to legalization and increased accessibility in the United States. With increasing usage, it is crucial to determine if its use is associated with complications and outcomes.

**Methods:**

The American Burn Association (ABA) Noncommercial Burn Research Dataset was queried for admissions between 2012 and 2021 with drug screens positive for cannabis. The primary outcome investigated was mortality. Secondary outcomes examined were ICU admission, respiratory failure, pneumonia, hospital length of stay (LOS), renal failure, unplanned intubation, arrhythmia, cellulitis, systemic sepsis, and cardiac arrest. Associations with hospital LOS were modeled with Ordinary Least Squares (OLS) regression. Multivariate logistic regression analysis described associations between cannabis drug screen positivity and all other primary outcomes. Covariates – age, sex, TBSA%, and inhalation injury – were included in all regression analyses to remove the effects of major confounders. Secondary complications odds ratios (OR) were calculated with the chi-square test. All data analysis was performed within PyCharm 3.1 software using pandas, NumPy, and Scipy.stats modules.

**Results:**

Out of the 51,238 patients with a drug screen, 11,693 tested positive for cannabis (22.8%). In 2012, the positivity rate for cannabis was 22.3%, which then doubled to 45.8% in 2021. Patients who tested positive for cannabis were significantly more likely to be male (p < 0.0001), younger (37.41±14.99 vs. 42.53±16.11, p < 0.0001), and have a scald injury (p < 0.0001). When adjusting for covariates, cannabis positivity was associated with a significantly decreased rate of ICU admissions (OR = 0.70, p < 0.0001) and a shorter hospital LOS (0.71 days, p < 0.001). All other complications and outcomes did not demonstrate any statistically significant differences for those with positive and negative cannabis drug screens.

**Conclusions:**

The prevalence of cannabis users in burn unit admissions doubled from 2012 to 2021, likely due to legalization and ease of access. Despite its augmented use, cannabis is not associated with increased rates of complications. Instead, those who test positive for cannabis have significantly lower rates of ICU admission and shorter hospital LOS. This potentially is due to less severe injuries and less depth; however, our dataset did not capture this metric. Further research is needed to investigate the effects of cannabis use on burn injuries and their management.

**Applicability of Research to Practice:**

Further research is needed to investigate the effects of cannabis use on burn injuries and their management.

**Funding for the Study:**

N/A

